# Multiple Synchronous Outbreaks of Puumala Virus, Germany, 2010

**DOI:** 10.3201/eid1809.111447

**Published:** 2012-09

**Authors:** Jakob Ettinger, Jorg Hofmann, Martin Enders, Friedemann Tewald, Rainer M. Oehme, Ulrike M. Rosenfeld, Hanan Sheikh Ali, Mathias Schlegel, Sandra Essbauer, Anja Osterberg, Jens Jacob, Daniela Reil, Boris Klempa, Rainer G. Ulrich, Detlev H. Kruger

**Affiliations:** Charité Medical School and Labor Berlin Charité-Vivantes GmbH, Berlin, Germany (J. Ettinger, J. Hofmann, B. Klempa, D.H. Kruger);; Institute of Virology, Infectious Diseases, and Epidemiology, Stuttgart, Germany (M. Enders, F. Tewald);; Baden-Wuerttemberg State Health Office, Stuttgart (R.M. Oehme);; Friedrich-Loeffler Institut, Greifswald, Germany (U.M. Rosenfeld, H.S. Ali, M. Schlegel, R.G. Ulrich);; Bundeswehr Institute of Microbiology, Munich, Germany (S. Essbauer, A. Osterberg);; Institute for Plant Protection in Horticulture and Forestry, Münster, Germany (J. Jacob, D. Reil);; and Slovak Academy of Sciences, Bratislava, Slovakia (B. Klempa)

**Keywords:** hantavirus outbreak, hemorrhagic fever with renal syndrome, HFRS, Puumala virus, hantavirus, viruses, molecular epidemiology, Germany

## Abstract

To investigate 2,017 cases of hantavirus disease in Germany, we compared 38 new patient-derived Puumala virus RNA sequences identified in 2010 with bank vole–derived small segment RNA sequences. The epidemic process was driven by outbreaks of 6 Puumala virus clades comprising strains of human and vole origin. Each clade corresponded to a different outbreak region.

Human hantavirus disease is manifested as hemorrhagic fever with renal syndrome (HFRS) in Asia, Europe, and most probably Africa (*1*). Since 2001, laboratory-confirmed cases of HFRS in Germany, by law, must be reported by local health authorities to the Robert Koch Institute in Berlin, the central federal institution responsible for disease control and prevention. Approximately 200 HFRS cases/year are reported in nonepidemic years (incidence 0.25 cases/100,000 persons).

Local outbreaks of HFRS were reported in 2004–2005, a large outbreak with 1,688 cases was reported in 2007, and 2,017 cases were reported in 2010 (*2*–*4*). In 2010, the total HFRS incidence in Germany increased to 2.47 cases/100,000 persons. This increase was caused by outbreaks in specific regions; administrative districts in these regions reported incidences of <80 cases/100,000 persons (Technical Appendix). In contrast to previous outbreaks (*5*), the 2010 outbreak did not lead to increased incidence of HFRS being reported in countries neighboring Germany, such as Belgium (www.wiv-isp.be/epidemio/labo) and France (www.invs.sante.fr/fr/Dossiers-thematiques/Maladies-infectieuses/Zoonoses), which indicated the distinctive features of this outbreak in Germany.

Although 3 hantaviruses, Puumala virus (PUUV), Dobrava-Belgrade virus, and Tula virus circulate in rodent hosts in Germany and can infect humans (*6*–*8*), most hantavirus infections in humans are caused by PUUV. The natural reservoir of PUUV, the bank vole (*Myodes glareolus*), is widely distributed in Germany and other countries in Europe. Bank vole abundance fluctuates every few years with ≈3–4 years between maximum peak densities of up to several hundred animals per hectare (*9*). Years with high vole densities and increased numbers of registered human hantavirus infections were preceded by years of intense coverage with beech mast (nuts of the European beech tree [*Fagus sylvatica*]) (*10*).

Diagnosis of hantavirus infection in humans is usually based on serologic testing. Since 1994, only 22 human-derived PUUV sequences have been identified in Germany. We conducted molecular epidemiologic analysis of the 2010 outbreak in Germany by compiling 38 new PUUV sequences of human origin, which were compared with bank vole–derived small (S) segment RNA sequences from the different outbreak regions.

## The Study

We established a countrywide alert network that included several physicians and diagnostic laboratories. This network enabled assessment of serum samples from patients during the early clinical phase of infection.

A total of 491 serum samples were tested for antibodies to hantavirus. Of these samples, 377 were positive for IgG against PUUV, of which 330 were positive for IgM against PUUV by an in-house ELISA (*11*) and immunoblot assay (*recom*Line Bunyavirus; Mikrogen, Neuried, Germany). These acute-phase antibody-positive samples were then screened by using a nested pan hantavirus reverse transcription PCR (*12*) specific for a conserved region within the polymerase gene (large genomic segment). Using this approach, we identified 102/330 IgM- and IgG-positive samples that were PCR positive. Sequencing of this large segment region confirmed infection by PUUV.

To differentiate virus strains within the PUUV species, a higher variable nucleotide sequence was targeted by amplification of a 504-nt fragment of the genomic S segment encoding the nucleocapsid protein (*13*). Using this approach, we found that 38 of 102 PUUV-positive samples were S segment positive. Nucleotide sequences of these 38 samples were determined and used for molecular phylogenetic analysis. The dataset also included sequences obtained from human and *M. glareolus* vole samples collected in previous years and from voles captured during the 2010 outbreak. Rodent trapping was performed after permission was obtained from Federal State authorities (permit no. NRW 20.09.210, BW 35-9185.82/0261).

**Figure 1:**
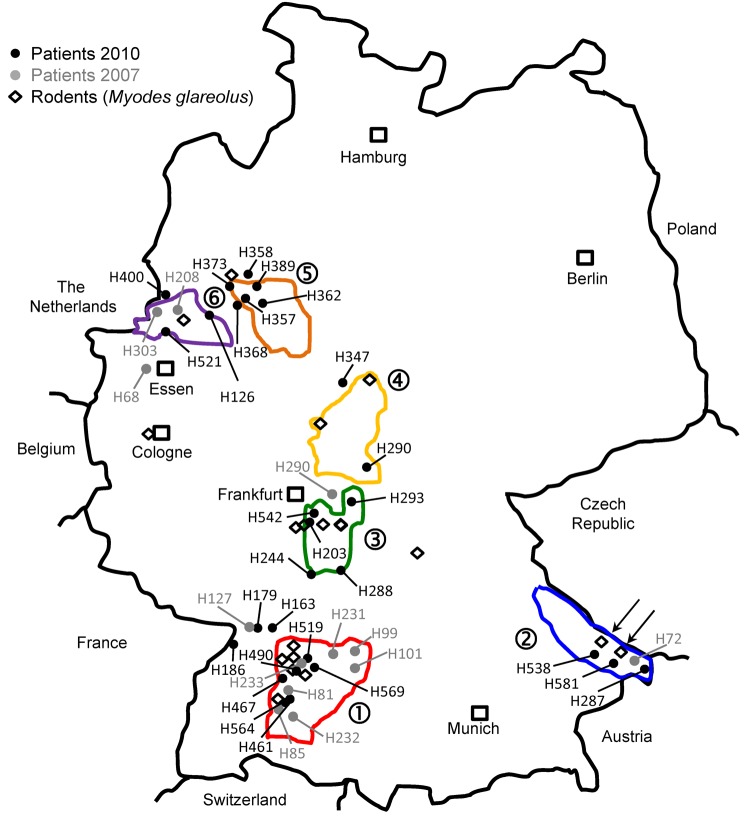
Distribution of investigated Puumala virus infections in Germany. Black dots indicate sequences obtained from patient samples in 2010; gray dots indicate sequences obtained from patient samples in 2007; diamonds indicate sequences obtained from rodent (*Myodes glareolus*) samples. Areas surrounded by lines indicate outbreak regions (numbered 1–6) where Puumala virus nucleotide sequences of human and vole origin have been analyzed. Numbers of the outbreak regions/virus clades and designations of local virus strains are also used in Figure 2. Arrows in outbreak region no. 2 indicate trapping sites of rodents from which strains 10 MuEb14v and 10 MuEb51 were obtained, which originated from localities 25 km apart.

**Figure 2:**
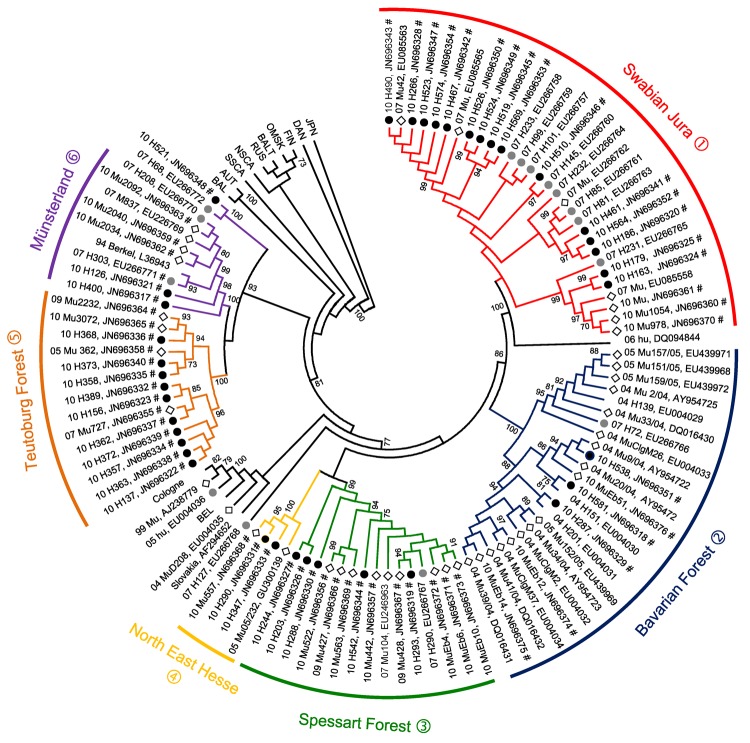
Neighbor-joining phylogenetic tree (TN93 evolutionary model) of Puumala virus (PUUV) strains constructed on the basis of partial sequences of the small segment (504-nt sequence, nt positions 392–894). Bootstrap values >70%, calculated from 10,000 replicates, are shown at the tree branches. Analysis was performed by using MEGA5 software (www.megasoftware.net). PUUV-like sequences from Japan (JPN) were used as outgroup. Numbers from 04 to 11 in front of the sample names indicate the year (2004–2011) when the sample was collected. Black dots indicate human samples from 2010, gray dots indicate human samples from 2007, and diamonds indicate rodent samples. Novel sequences from this study are indicated by the symbol #. For numbers (1–6) of PUUV clades that correspond to the 6 defined outbreak regions, see Figure 1. Phylogenetic clades are shown in parentheses followed by names and numbers. For clarity, previously characterized PUUV clades from other parts of Europe are shown in simplified form. BEL, Belgium; BAL, Balkan; AUT, Austrian; SSCA, South Scandinavian; NSCA, North Scandinavian; RUS, Russian; BALT, Baltic; OMSK, Russian from Omsk region; FIN, Finnish; DAN, Danish.

Residences of patients in Germany from whom viral sequences were obtained and corresponding rodent trapping sites are shown in Figure 1. Results of molecular phylogenetic analysis of these strains are shown in Figure 2. In addition to 4 molecular clades (Swabian Jura, clade 1; Bavarian Forest, clade 2; Spessart Forest, clade 3; and Münsterland, clade 6) found during the 2007 outbreak, 2 novel clades (North East Hesse, clade 4; and Teutoburg Forest, clade 5) were defined. All 6 clades comprised human-derived and vole-derived sequences.

Identification of new PUUV clades in 2010 was associated with a higher incidence of human disease than in 2007. For example, in the Federal State of Hesse, incidence increased from 0.35 cases/100,000 persons in 2007 to 2.80 cases/100,000 persons in 2010. This increase in cases enabled us to collect blood specimens from patients shortly after clinical onset of disease, characterize these specimens, and define a new PUUV clade (North East Hesse, clade 4).

To analyze diversity within and between different clades, we aligned each clade and calculated mean pairwise amino acid and nucleotide identities. In addition, we identified the consensus sequence of each clade and compared each with those of other clades (Table). Sequences within 1 clade show pairwise amino acid identities >97% and nucleotide identities >96%. Identities between clades range from 90.3% (Swabian Jura vs. Spessart Forest) to 97.7% (Münsterland vs. Teutoburg Forest) on the amino acid level and 81.3% (Teutoburg Forest vs. Swabian Jura) to 89.0% (Münsterland vs. Teutoburg Forest) on the nucleotide sequence level.

**Table:** Amino acid and nucleotide sequence identity rates within and between different Puumala virus clades, Germany*

Clade	No. samples	% Amino acid identity†	% Nucleotide identity†	% Identity					
				SJ (1)	BF (2)	SF (3)	NEH (4)	TF (5)	ML (6)
SJ (1)	30	97.9	96.2		91.5	90.3	91.5	90.9	92.6
BF (2)	24	99.3	97.9	85.7		95.2	96.3	95.2	96.8
SF (3	15	99.3	97.0	84.0	87.2		96.0	93.7	95.4
NEH (4)	4	99.3	96.9	83.9	85.8	86.7		95.4	97.1
TF (5)	13	99.4	98.2	81.3	84.4	83.3	84.8		97.7
ML (6)	9	100	98.7	83.1	84.7	83.1	84.4	89.0	

*Sequences of the 504-bp small segment were compared. Values above the diagonal indicate amino acid identity, and values below the diagonal indicate nucleotide identity. Numbers in parentheses indicate outbreak regions as shown in Figure 1 and Figure 2. SJ, Swabian Jura; BF, Bavarian Forest; SF, Spessart Forest; NEH, North East Hesse; TF, Teutoburg Forest; ML, Münsterland. †Within each clade.

Within a particular molecular clade, virus strains from localities near each other could be differentiated. For example, within PUUV strains from the Bavarian Forest, which has been known as an outbreak region since 2004 (*13*,*14*), newly characterized strains 10 Mu Eb51 and 10 Mu Eb14 (Bavarian Forest; Figure 1, Figure 2) originated from localities only 25 km apart. Because migration distances >1 km for bank voles are efficiently prevented by natural and artificial barriers (*15*), local vole populations are likely associated with specific PUUV strains.

## Conclusions

Three years after a large 2007 PUUV outbreak, a subsequent epidemic with >2,000 human cases occurred in Germany. On the basis of beech mast coverage in 2009, growth of the reservoir rodent population in 2010 was expected. Model monitoring studies in July 2010 found mean ± SD bank vole densities of <141 ± 13 voles/hectare in investigated outbreak regions. PUUV seroprevalence in these bank vole populations was >40%. Bank vole densities in the same regions decreased to <15 voles/hectare in April 2011 (D. Reil, U.M. Rosenfeld, unpub. data).

Phylogenetic analysis of the involved PUUV strains identified 6 clades comprising virus strains of human and vole origin. Each clade clearly corresponds to a different outbreak region. High molecular similarities of human- and rodent-derived PUUV sequences from the same geographic origin and their clear molecular distinction from viruses of neighboring regions indicate spatial evolution of each virus clade. Thus, we conclude that the 2010 epidemic was not caused by countrywide spread of the same virus but resulted from multiple local outbreaks associated with simultaneous increases in densities and infection rates of bank voles in the different geographic regions.

At least for the investigated 504-nt S segment region, no evidence was found that viruses had undergone changes over time or as a result of rodent-to-human transmission. Lack of human-to-human transmission, local distribution of bank voles, and a high degree of identity between viruses of human and rodent origin enabled us to allocate certain virus strains to defined geographic regions. Future investigations may enable generation of risk maps with higher resolution and establishment of more sophisticated preventive measures in high-risk areas.

Technical Appendix Incidence of hantavirus disease in outbreak regions and during outbreak year (2010) and preoutbreak and postoutbreak years, Germany.
