# Supplementary material for: Multiple Synchronous Outbreaks of Puumala Virus, Germany, 2010
Source: Emerg Infect Dis. 2012 Sep;18(9):1461–4. doi: 10.3201/eid1809.111447 (PMC3437711; doi:10.3201/eid1809.111447)
Supplement: Technical Appendix — Incidence of hantavirus disease in outbreak regions and during outbreak year (2010) and preoutbreak and postoutbreak years, Germany. [file 11-1447-Techapp-s1.pdf]

Article DOI: http://dx.doi.org/10.3201/eid1809.111447

Multiple Synchronous Outbreaks of Puumala Virus, Germany, 2010

Technical Appendix

Technical Appendix Table. Incidence of hantavirus disease in outbreak regions and during outbreak year (2010) and preoutbreak and postoutbreak years, Germany*

Outbreak region, administrative district	Mean incidence/100,000 persons		
	2009	2010	2011
Germany, NA	0.22	2.47	0.37
Swabian Jura			
Alb-Donau-Kreis	2.11	21.62	2.64
Boeblingen	1.61	26.12	0.27
Donau-Ries	2.32	10.90	1.55
Enzkreis	2.57	21.66	2.58
Esslingen	0.58	11.46	2.72
Goeppingen	4.73	18.61	6.34
Heidenheim	3.03	16.78	3.81
Ludwigsburg	0.19	7.92	0.19
Pforzheim	0.00	15.03	0.83
Rems-Murr-Kreis	0.24	2.41	0.72
Reutlingen	2.49	40.22	3.92
Sigmaringen	1.53	18.43	5.38
Stuttgart	1.66	27.37	1.48
Tübingen	1.36	11.75	1.36
Zollernalbkreis	1.06	10.09	2.12
Bavarian Forest			
Cham	0.00	0.78	0.00
Deggendorf	0.86	14.53	2.56
Freyung-Grafenau	3.76	52.97	6.31
Passau	0.00	4.80	1.07
Regen	1.26	8.87	3.80
Spessart			
Aschaffenburg (rural)	0.58	31.85	2.32
Aschaffenburg (urban)	0.00	14.56	1.46
Bad Kissingen	0.95	34.52	1.92
Main Spessart	1.55	80.62	4.70
Main Tauber	0.75	9.00	0.75
Miltenberg	0.77	28.05	0.00
Neckar-Odenwald-Kreis	2.03	23.13	1.36
Rhön-Grabfeld	0.00	13.27	1.21
Schweinfurt	0.00	4.42	0.00
Schweinfurt	0.00	1.87	0.00
Wuerzburg (rural)	2.50	20.65	2.50
Wuerzburg (urban)	0.75	10.46	3.74
North East Hesse			
Fulda	0.00	16.11	0.00
Hersfeld-Rotenburg	0.00	9.82	1.64
Schwalm-Eder-Kreis	0.54	6.57	1.10
Vogelsberkreis	0.90	9.14	0.91
Werra-Meißner-Kreis	0.00	3.86	0.00
Teutoburg Forest			
Bielefeld	0.31	0.62	0.00
Guetersloh	0.28	0.57	0.00
Lippe	0.00	2.56	0.28
Osnabrueck (rural)	1.83	7.31	0.00
Osnabrueck (urban)	1.40	21.62	1.40
Paderborn	1.00	1.00	0.67
Munsterland			
Borken	1.35	3.25	2.43
Coesfeld	1.36	6.37	1.82
Muenster	1.09	5.72	1.43

Outbreak region, administrative district	Mean incidence/100,000 persons		
	2009	2010	2011
Steinfurt	0.45	1.35	0.68
Warendorf	0.00	1.08	0.72

*Source: Robert Koch-Institut, Federal Institute for Disease Control and Prevention (www3.rki.de/SurvStat). Data were obtained on March 23, 2012. NA, not applicable.
